# Neuroinflammation After COVID-19 With Persistent Depressive and Cognitive Symptoms

**DOI:** 10.1001/jamapsychiatry.2023.1321

**Published:** 2023-05-31

**Authors:** Joeffre Braga, Mariel Lepra, Stephen J. Kish, Pablo. M. Rusjan, Zahra Nasser, Natasha Verhoeff, Neil Vasdev, Michael Bagby, Isabelle Boileau, M. Ishrat Husain, Nathan Kolla, Armando Garcia, Thomas Chao, Romina Mizrahi, Khunsa Faiz, Erica L. Vieira, Jeffrey H. Meyer

**Affiliations:** 1 Brain Health Imaging Centre, Campbell Family Mental Health Research Institute, The Centre for Addiction and Mental Health, Toronto, Ontario, Canada; 2 Azrieli Centre for Neuro-Radiochemistry, Campbell Family Mental Health Research Institute, The Centre for Addiction and Mental Health Toronto, Ontario, Canada; 3 Department of Pharmacology and Toxicology, University of Toronto, Toronto, Ontario, Canada; 4 Department of Psychiatry, University of Toronto, Toronto, Ontario, Canada; 5 Douglas Research Centre, McGill University, Montreal, Quebec, Canada; 6 Department of Psychiatry, McGill University, Montreal, Quebec, Canada; 7 Department of Psychology, University of Toronto, Toronto, Ontario, Canada; 8 Waypoint Centre for Mental Health Care, Penetanguishene, Ontario, Canada; 9 Department of Psychiatry, Institute of Mental Health, University of British Columbia, Vancouver, Canada; 10 Department of Diagnostic Radiology, Hamilton Health Sciences, McMaster University, Hamilton, Ontario, Canada

## Abstract

**Importance:**

Persistent depressive symptoms, often accompanied by cognitive symptoms, commonly occur after COVID-19 illness (hereinafter termed *COVID-DC*, DC for depressive and/or cognitive symptoms). In patients with COVID-DC, gliosis, an inflammatory change, was suspected, but measurements of gliosis had not been studied in the brain for this condition.

**Objective:**

To determine whether translocator protein total distribution volume (TSPO V_T_), a marker of gliosis that is quantifiable with positron emission tomography (PET), is elevated in the dorsal putamen, ventral striatum, prefrontal cortex, anterior cingulate cortex, and hippocampus of persons with COVID-DC.

**Design, Setting, and Participants:**

This case-control study conducted at a tertiary care psychiatric hospital in Canada from April 1, 2021, to June 30, 2022, compared TSPO V_T_ of specific brain regions in 20 participants with COVID-DC with that in 20 healthy controls. The TSPO V_T_ was measured with fluorine F 18–labeled *N*-(2-(2-fluoroethoxy)benzyl)-*N*-(4-phenoxypyridin-3-yl)acetamide ([^18^F]FEPPA) PET.

**Main Outcomes and Measures:**

The TSPO V_T_ was measured in the dorsal putamen, ventral striatum, prefrontal cortex, anterior cingulate cortex, and hippocampus. Symptoms were measured with neuropsychological and psychological tests, prioritizing outcomes related to striatal function.

**Results:**

The study population included 40 participants (mean [SD] age, 32.9 [12.3] years). The TSPO V_T_ across the regions of interest was greater in persons with COVID-DC (mean [SD] age, 32.7 [11.4] years; 12 [60%] women) compared with healthy control participants (mean [SD] age, 33.3 [13.9] years; 11 [55%] women): mean (SD) difference, 1.51 (4.47); 95% CI, 0.04-2.98; 1.51 divided by 9.20 (17%). The difference was most prominent in the ventral striatum (mean [SD] difference, 1.97 [4.88]; 95% CI, 0.36-3.58; 1.97 divided by 8.87 [22%]) and dorsal putamen (mean difference, 1.70 [4.25]; 95% CI, 0.34-3.06; 1.70 divided by 8.37 [20%]). Motor speed on the finger-tapping test negatively correlated with dorsal putamen TSPO V_T_ (*r*, −0.53; 95% CI, −0.79 to −0.09), and the 10 persons with the slowest speed among those with COVID-DC had higher dorsal putamen TSPO V_T_ than healthy persons by 2.3 (2.30 divided by 8.37 [27%]; SD, 2.46; 95% CI, 0.92-3.68).

**Conclusions and Relevance:**

In this case-control study, TSPO V_T_ was higher in patients with COVID-DC. Greater TSPO V_T_ is evidence for an inflammatory change of elevated gliosis in the brain of an individual with COVID-DC. Gliosis may be consequent to inflammation, injury, or both, particularly in the ventral striatum and dorsal putamen, which may explain some persistent depressive and cognitive symptoms, including slowed motor speed, low motivation or energy, and anhedonia, after initially mild to moderate COVID-19 illness.

## Key Points

**Question:**

Is translocator protein distribution volume (TSPO V_T_), an index of gliosis (an inflammatory change), measured by positron emission tomography, elevated in the brain after acute COVID-19 infection with sequelae of depressive and cognitive symptoms?

**Findings:**

In this case-control study, TSPO V_T_ was elevated in 20 participants with persistent depressive and cognitive symptoms after initially mild to moderate COVID-19 infection when compared with 20 healthy controls, more prominently in the ventral striatum and dorsal putamen. The TSPO V_T_ in the dorsal putamen of COVID-19 cases negatively correlated with motor speed.

**Meaning:**

These findings suggest that gliosis, especially in the ventral striatum and dorsal putamen, may reflect injury, ongoing inflammation, or both and provide directions for further therapeutic development.

## Teaser

This case-control study uses positron emission tomography to compare translocator protein distribution volume, a quantitative marker of gliosis, in brain regions of interest between healthy persons and persons with persistent depressive and cognitive symptoms after mild to moderate acute COVID-19 infection.

## Introduction

Documented acute COVID-19 infection affects about a third of the population in countries where this is recorded, but it is generally believed that the majority of the world’s population has experienced at least 1 acute episode of COVID-19 illness.^1^ During acute COVID-19 illness, mild to moderate symptoms occur in approximately 95% of people infected with an Omicron variant^2^ and in approximately 80% of people infected with the initial SARS-CoV-2 strain.^3^ Mild severity indicates the presence of some symptoms, whereas moderate severity includes shortness of breath not needing supplemental oxygen.^4^ Several days to months after acute symptoms abate, there is a high prevalence of depressive symptoms with or without cognitive impairment, ranging from approximately 15% after wild-type SARS-CoV-2 exposure^5^ in unvaccinated persons to approximately 5% in triple-vaccinated persons exposed to Delta or Omicron variants.^2^ Many of these symptoms, such as anhedonia, motor slowing, low motivation and energy, and short-term memory impairment may persist for months to years.^6,7,8^ Hence, depressive symptoms with or without other cognitive symptoms, after an acute episode of mild to moderate COVID-19 illness, hereinafter termed *COVID-DC*, is a major public health problem.

There are several reasons why gliosis may be present in COVID-DC. First, gliosis is implicated as an etiological mechanism for depressive and cognitive symptoms across neuropsychiatric diseases through a number of processes, including the generation of cytokines, prostaglandins, reactive nitrogen species, and reactive oxygen species.^9^ Second, some events that occur during acute COVID-19 illness may promote brain gliosis, such as elevation in peripheral cytokines signaling brain inflammation,^10^ new-onset hypoxic or vascular occlusion lesions, or possibly viral uptake into the brain.^11^ Third, half of postmortem studies collecting tissue specimens from persons with severe to critical acute COVID-19 illness leading to death report gliosis with microglial or astroglial activation in the brain regions sampled.^12,13^ Although these arguments are persuasive, they have limitations, most notably that gliosis in postmortem samples taken during severe to critical acute COVID-19 illness is not the equivalent of gliosis obtained during COVID-DC^12,13^: samples collected from patients during acute COVID-19 illness are obtained at a time of widespread bodily infection and months earlier than the time of COVID-DC. Hence, patients whose samples are collected during acute COVID-19 have not yet had lengthy depressive or cognitive symptoms. In addition, postmortem samples collected from persons with acute COVID-19 illness reflect severe to critical disease, in contrast to the vast majority of clinical COVID-19 cases, which are mild to moderate.^2,3^ In the present study, we sampled cases with this latter clinical history. Persons with a history of mild to moderate COVID-19 disease would be unlikely to experience certain phenomena seen in severe cases, such as the cytokine storm associated with highly elevated, generalized bodily inflammation with dysregulation of clotting factors and greater risk for vascular lesions or, since supplemental oxygen is not required, hypoxic lesions. Thus, while there is reason to suspect gliosis in COVID-DC, it is unknown whether gliosis is present in the brains of individuals with COVID-DC.

Positron emission tomography (PET) imaging of the translocator protein (TSPO) can detect gliosis composed of activated microglia or astroglia.^14^ During health, most TSPO binding is attributable to its presence in endothelial cells; however, in neuropsychiatric diseases with brain inflammation, most TSPO binding is associated with microglia and, to a lesser extent, astroglia transitioned or transitioning to an activated state.^14^ Fluorine F 18–labeled *N*-(2-(2-fluoroethoxy)benzyl)-*N*-(4-phenoxypyridin-3-yl)acetamide ([^18^F]FEPPA) is an excellent radiotracer for PET measurement of TSPO total distribution volume (V_T_), with high selectivity, high affinity, high brain uptake, and no brain-penetrant radioactive metabolites.^15,16^ Moreover, [^18^F]FEPPA has been modeled in humans,^15^ and TSPO V_T_, an index of TSPO density and a marker of gliosis, is elevated in diseases known to be associated with gliosis, such as Alzheimer disease.^17^

The main objective of this study was to compare TSPO V_T_ in the dorsal putamen, ventral striatum, prefrontal cortex, anterior cingulate cortex, and hippocampus of persons with COVID-DC vs healthy controls, and it was hypothesized that TSPO V_T_ would be elevated in persons with COVID-DC. These regions were chosen because injury in these regions, which can cause gliosis, also induces symptoms of COVID-DC. The ventral striatum and dorsal putamen were chosen for their respective roles in modulating reward,^18,19^ motivation, and movement speed, functions often affected in COVID-DC.^6,7,8^ The prefrontal cortex and anterior cingulate cortex were chosen for their roles in regulating affect as demonstrated in mood induction studies^20^ and the hippocampus for its impact on cognitive functioning (eMethods in Supplement 1). Given these roles of these brain regions, the following associations are hypothesized between function and TSPO V_T_: slower motor speed associated with the dorsal putamen; severity of depression symptoms associated with both the prefrontal cortex and anterior cingulate cortex; and cognitive concerns associated with the hippocampus.

## Methods

### Participants

This study was conducted from April 1, 2021, to June 30, 2022. There were 40 participants aged 18 to 72 years who completed the PET imaging protocol. In total, 20 participants had COVID-DC and were compared with 20 healthy controls, the latter selected for their matching rs6971 genotype, which affects radiotracer binding to TSPO. Controls of similar age and sex (Table 1) were recruited prior to the COVID-19 pandemic, between 2009 and 2018. Participants were recruited from the southern Ontario, Canada, region through clinics at the Centre for Addiction and Mental Health and through advertisement (eMethods in Supplement 1). All participants provided written informed consent. The protocol and informed consent forms were approved by the Research Ethics Board of the Centre for Addiction and Mental Health in Toronto, Ontario, Canada, and by Health Canada. This study followed the Strengthening the Reporting of Observational Studies in Epidemiology (STROBE) reporting guideline.

**Table 1.** Baseline Characteristics of Study Participants

Characteristic^a^	Participants, No. (%)	
	COVID-DC (n = 20)	Healthy controls (n = 20)
Sex		
Female	12 (60)	11 (55)
Male	8 (40)	9 (45)
Age, mean (SD), y	32.7 (11.4)	33.3 (13.9)
Duration between COVID-19 diagnosis and PET scan, mo		
0-6	12 (60)	NA
7-24	8 (40)	NA
Acute COVID-19 severity		
Mild	3 (15)	NA
Moderate	17 (85)	NA
Neurological symptoms during acute COVID-19 illness		
Headache	14 (70)	NA
Loss of taste	11 (55)	NA
Loss of smell	11 (55)	NA
Confusion	8 (40)	NA
Ongoing neurological symptoms at time of PET scan		
Headache	6 (30)	NA
Loss of ability to use speech	0 (0)	NA
Loss of ability to move	0 (0)	NA
Loss of sensation in a body part	0 (0)	NA
Confusion	3 (15)	NA
Ongoing physical symptoms at time of PET scan		
Nasal congestion or runny nose	4 (20)	NA
Tiredness	12 (60)	NA
Aches or pains	4 (20)	NA
Muscle aches or pains	4 (20)	NA
17-item HDRS score, mean (SD)	17.7 (5.3)^b^	NA
Past MDE prior to COVID-19 infection	9 (45)	NA
BMI, mean (SD)	26.2 (3.8)	25.3 (4.0)
TSPO genotype		
HAB^c^	18 (90)	18 (90)
MAB^c^	2 (10)	2 (10)

Abbreviations: BMI, body mass index (calculated as weight in kilograms divided by height in meters squared); COVID-DC, COVID-19 and depression with or without other cognitive symptoms; HAB, high-affinity binder; HDRS, Hamilton Depression Rating Scale; MAB, mixed-affinity binder; MDE, major depressive episode; NA, not applicable; PET, positron emission tomography, TSPO, translocator protein.

^a^ Some rows with 1 or 2 participants have been amalgamated.

^b^ Scores derived on the day of scanning.

^c^ HAB and MAB based on rs6971 genotype.

To meet minimum illness severity, the main inclusion criterion for participants with COVID-DC was onset of a new major depressive episode (MDE), documented with the Structured Clinical Interview for *DSM-5*—Research Version,^21^ within 3 months of acute COVID-19 illness verified by polymerase chain reaction or rapid antigen testing. Previous acute COVID-19 illness was required to be of mild or moderate severity^4,22^ as documented with structured questionnaires (eMethods in Supplement 1). The main inclusion criteria for healthy participants were good health, based on answers to a structured health questionnaire, and no history of psychiatric illness documented with the Structured Clinical Interview for *DSM-5*—Research Version.^21^ Exclusion criteria common to all cases were no history of neurological disease prior to COVID-19 infection, no use of anti-inflammatory medication within the previous 4 weeks (including nonsteroidal anti-inflammatory medication), current substance use disorder, lifetime history of moderate or severe substance use disorder, and cigarette or substance use within the previous 2 months (including marijuana), the latter further verified by urine drug screen (eMethods in Supplement 1).

### PET and Magnetic Resonance Imaging Acquisition and Image Analysis

A 3-dimensional high-resolution research tomograph (CPS/Siemens) PET scanner acquired imaging data for 120 minutes as previously described^23^ (eMethods in Supplement 1). During the emission PET scan, arterial sampling was done using an automatic blood sampling system and manual sampling. For determining of regions of interest (eMethods in Supplement 1), T1-weighted brain magnetic resonance images (Discovery MR750 3.0-T scanner (GE Healthcare) with an 8-channel head coil) were acquired. The TSPO V_T_ data were calculated using a 2-tissue compartment model previously validated for [^18^F]FEPPA PET.^23^

### Assessment of Persistent Symptoms After COVID-19 Infection

In addition to the Structured Clinical Interview for *DSM-5*—Research Version,^21^ participants with COVID-DC completed neuropsychological and psychological testing by trained staff (eTable 1 in Supplement 1). Priority measures included motor speed with the finger-tapping test,^24^ overall severity of MDE based on the 17-item Hamilton Depression Rating Scale (HDRS) score (range, 0 to 52, with a higher score indicating greater overall severity of an MDE),^25^ and magnitude of self-perceived deficits in cognitive functioning based on the Cognitive Failures Questionnaire (CFQ) score (range, 0 to 100, with a higher score indicating more cognitive difficulties relative to demands of environment).^26^

### Statistical Analysis

Initial analyses compared TSPO V_T_ between participants with COVID-DC and healthy controls by applying repeated-measures analysis of variance (ANOVA) and mixed-effects models evaluating group and region, and rs6971 genotype as factors or fixed effects, respectively, and in the case of mixed-effects models, participant as a random effect. Where the statistical significance of repeated-measures ANOVA and mixed-effects models was the same, 1 *P* value is reported. For the prioritized main analysis, for samples of 20 in each group, this study had an 80% power to detect a 20% difference in TSPO V_T_ between COVID-DC and controls (eMethods in Supplement 1). An exploratory voxel-based analysis comparing persons with COVID-DC and controls using an ANOVA, assessing the association of group, with genotype as a factor, was also conducted (eMethods in Supplement 1).

The association between function and regional TSPO V_T_ (corrected for rs6971 genotype) was assessed using Pearson correlation analysis as follows: age- and sex-corrected T-scores on the finger-tapping test with dorsal putamen TSPO V_T_; HDRS score with prefrontal cortex and anterior cingulate cortex TSPO V_T_; and CFQ score with hippocampus TSPO V_T_ (TSPO V_T_ data for 4 persons who were mixed-affinity binders were adjusted by a factor of 1.4,^27,28^ eMethods in Supplement 1). A 2-sided value of *P* < .05 was considered statistically significant. All data were analyzed with SPSS, version 25 (SPSS-IBM).

## Results

In total, 40 participants (mean [SD] age, 32.9 [12.3] years), 20 with COVID-DC (mean [SD] age, 32.7 [11.4] years; 12 [60%] women and 8 men [40%]) and 20 healthy controls (mean [SD] age, 33.3 [13.9] years; 11 [55%] women and 9 [45%] men) were included in the analyses (Table 1; eMethods in Supplement 1). Prominent symptoms of COVID-DC were anhedonia (n = 20), motor speed slowing (n = 19 with age- and sex-corrected T-scores below 50; 14 participants were at impairment-level severity with T-scores below 40; tests of 1 participant with past training related to cognitive testing were excluded; and 1 participant was unable to complete tests with the nondominant hand due to mild injury in arm), energy problems largely attributed to low motivation (n = 18), and cognitive concerns (16 participants scoring above 33 on the CFQ, indicating an elevated level of cognitive concern relative to situational demands). Depressive, cognitive, and other common persisting symptoms are listed in Table 1.

**Figure 1.**
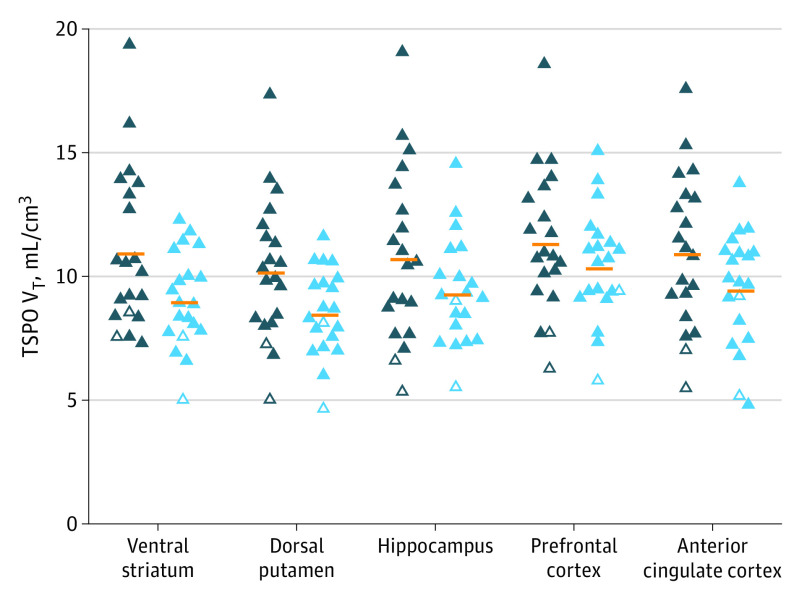
Translocator Protein Total Distribution Volume (TSPO V_T_) in Brain Regions of Interest in Persons With COVID-19 Disease and Depression With or Without Other Cognitive Symptoms (COVID-DC) vs Healthy Persons Closed triangles represent high-affinity binders; open triangles, mixed-affinity binders (MAB); dark blue symbols, 20 persons with COVID-DC; light blue symbols, 20 healthy persons; horizontal orange lines, group means. After correcting for genotype effect by multiplying MAB TSPO V_T_ by a factor of 1.4, nonparametric comparison with the Mann-Whitney *U *test yielded similar levels of statistical significance (ventral striatum, *U* = 117, *P* = .02; dorsal putamen, *U* = 121, *P* = .03; hippocampus, *U* = 156, *P* = .24; prefrontal cortex, *U* = 164, *P* = .34; anterior cingulate cortex, *U* = 143, *P* = .13).

Group analyses comparing TSPO V_T_ collectively across prioritized regions of the ventral striatum, dorsal putamen, prefrontal cortex, anterior cingulate cortex, and hippocampus (ie, including TSPO V_T_ for each region as data) found greater TSPO V_T_ in persons with COVID-DC (mean [SD] TSPO V_T_, 9.23 [3.16] mL/cm^3^ vs control mean [SD] TSPO V_T_, 7.72 [3.16] mL/cm^3^; mean [SD] difference, 1.51 [4.47; 95% CI, 0.04-2.98; *P* = .04]; 1.51 divided by 9.20 [17%]) (Figure 1, Table 2). Among those regions, ventral striatum (COVID-DC mean [SD] TSPO V_T_, 9.56 [3.45] mL/cm^3^; control mean [SD] TSPO V_T_, 7.59 [3.45] mL/cm^3^; mean [SD] difference, 1.97 [4.88; 95% CI, 0.36-3.58; *P* = .02]) and dorsal putamen (COVID-DC mean [SD] TSPO V_T_, 8.67 [3.01] mL/cm^3^; control mean [SD] TSPO V_T_, 6.97 [3.01] mL/cm^3^; mean [SD] difference, 1.70 [4.25; 95% CI, 0.34-3.06; *P* = .02]) values were most elevated compared with controls (Figure 1, Table 2, eTable 2 in Supplement 1).

**Table 2.** Regional Translocator Protein Total Distribution Volume in Participants After COVID-19 Illness and Depression With or Without Other Cognitive Symptoms and in Healthy Participants^a^

Region of interest	TSPO V_T_, mean (SD) mL/cm^3^		Between-group difference (95% CI)	*P* value
	COVID-DC (n = 20)	Healthy (n = 20)		
Ventral striatum	10.8 (3.2)	8.9 (1.9)	1.97 (0.36 to 3.58)	.02
Dorsal putamen	10.1 (2.8)	8.4 (1.8)	1.70 (0.34 to 3.06)	.02
Hippocampus	10.6 (3.5)	9.2 (2.1)	1.43 (−0.29 to 3.14)	.10
Anterior cingulate cortex	10.8 (3.1)	9.3 (2.3)	1.47 (−0.12 to 3.07)	.07
Prefrontal cortex	11.2 (2.9)	10.2 (2.2)	0.99 (−0.48 to 2.45)	.18
Dorsal caudate	9.3 (2.5)	8.0 (2.1)	1.29 (−0.15 to 2.73)	.08
Thalamus	13.2 (3.6)	11.5 (2.4)	1.72 (−0.08 to 3.51)	.06
Temporal cortex	11.6 (3.0)	10.4 (2.3)	1.19 (−0.39 to 2.77)	.14
Occipital cortex	11.8 (3.0)	10.6 (2.2)	1.19 (−0.33 to 2.72)	.12
Parietal cortex	12.0 (3.0)	11.0 (2.3)	1.06 (−0.48 to 2.60)	.17
Insula	11.2 (3.0)	9.9 (2.2)	1.35 (−0.15 to 2.84)	.08
Midbrain	11.8 (3.6)	11.3 (2.7)	0.55 (−1.36 to 2.46)	.56
Cerebellum	11.5 (3.1)	10.2 (2.2)	1.26 (−0.29 to 2.82)	.11

Abbreviations: COVID-DC, COVID-19 and depression with or without other cognitive symptoms; TSPO V_T_, translocator protein total distribution volume.

^a^ Repeated-measures analysis of variance with region as the repeated measure evaluating the association of group with all regions yielded statistically insignificant results (COVID-DC mean [SD] TSPO V_T_, 9.69 [3.18] mL/cm^3^; control mean [SD] TSPO V_T_, 8.48 [3.13] mL/cm^3^; mean [SD] difference, 1.21 [4.46]; 95% CI, −0.30 to 2.71; *P* = .11). Means presented in Table 2 are derived from raw unadjusted TSPO V_T_ values.

The percentage differences between COVID-DC and controls were 1.97 divided by 8.87 (22%) in the ventral striatum and 1.70 divided by 8.37 (20%) in the dorsal putamen, as calculated by applying the following equation: the COVID-DC mean minus the control mean divided by the control mean using the exact mean values given in Table 2. In the preceding paragraph, adjusted means from SPSS are reported, and SPSS used adjusted means that incorporate the effect of the factor for high-affinity binders vs mixed-affinity binders. Applying SPSS-adjusted means to the same equation yielded larger percentage differences of 1.97 divided by 7.59 (26%) in the ventral striatum and 1.70 divided by 6.97 (24%) in the dorsal putamen. All brain regions assayed had greater TSPO V_T_ in persons with COVID-DC than in healthy controls, but the magnitude and statistical significance level varied (Table 2). Region of interest results were fairly similar to those of the exploratory voxel-based analysis (eResults in Supplement 1).

**Figure 2.**
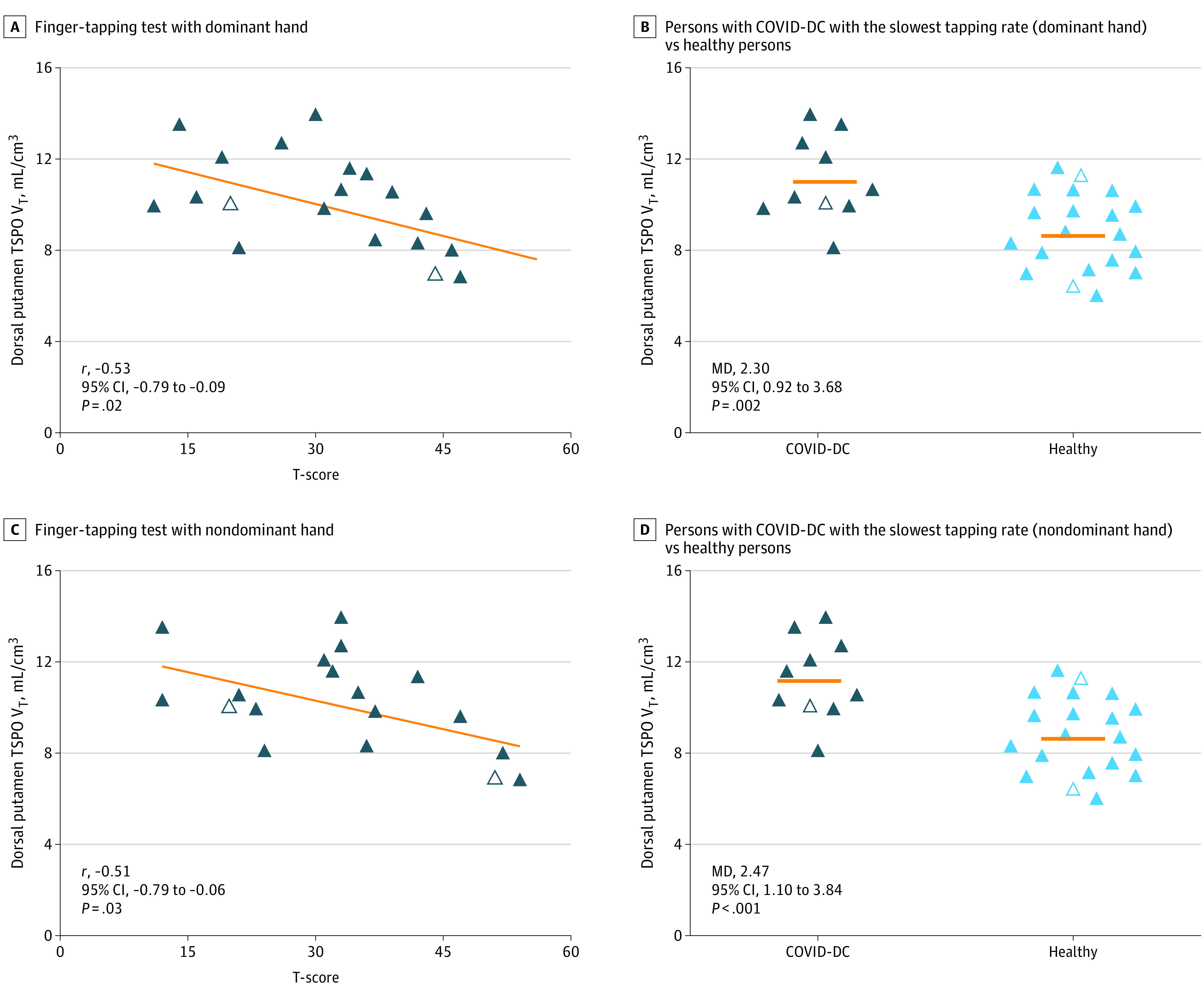
Pearson Correlation Between Translocator Protein Total Distribution Volume (TSPO V_T_) in the Dorsal Putamen and Motor Speed A, Negative correlation between TSPO V_T_ in dorsal putamen and T-score on a finger-tapping test with the dominant hand in 19 persons with COVID-19 illness and depression with or without other cognitive symptoms (COVID-DC). B, Comparison of TSPO V_T_ in dorsal putamen of 10 participants with COVID-DC with the slowest tapping rate with their dominant hand vs 20 healthy persons. C, Negative correlation between TSPO V_T_ in dorsal putamen with T-score on a finger-tapping test with the nondominant hand in 18 persons with COVID-DC. D, Comparison of TSPO V_T_ in dorsal putamen of 10 persons with COVID-DC with the slowest tapping with their nondominant hand vs 20 healthy persons. For all panels, TSPO V_T_ of mixed-affinity binders (n = 4 total) is multiplied by 1.4 to adjust for rs6971 genotype effect on radiotracer binding. Horizontal orange lines represent group means; closed triangles, high-affinity binders; open triangles, mixed-affinity binders; dark-blue symbols, persons with COVID-DC; light-blue symbols, healthy persons. MD indicates mean difference.

In participants with COVID-DC, greater TSPO V_T_ in the dorsal putamen was associated with slower motor speed measured with mean T-scores on the finger-tapping test (*r*, −0.53; 95% CI, −0.79 to −0.09; *P* = .02) (Figure 2). The 10 persons with the slowest speed among individuals with COVID-DC illness had higher mean (SD) dorsal putamen TSPO V_T_ than healthy persons by 2.3 (2.46; 95% CI, 0.92-3.68; 2.30 divided by 8.37 [27%]). The association of the ventral striatum TSPO V_T_ with the presence of anhedonia was not assessable because these symptoms were reported by all participants. There was no significant correlation between the HDRS score and the prefrontal cortex (*r*, −0.02; *P *= .95) or anterior cingulate cortex TSPO V_T_ (*r*, 0.17; *P *= .48), nor was there a significant correlation between the total CFQ score and hippocampal TSPO V_T_ (*r*, −0.17; *P *= .47).

Exploratory analyses found that the associations of regional TSPO V_T_ with other tests were largely nonsignificant (eTable 1 in Supplement 1). There were no significant correlations of the ventral striatum TSPO V_T_ or the dorsal putamen TSPO V_T_ with duration since acute COVID-19 illness. There was also no association between timing of predominant variants in Canada and the ventral striatum or dorsal putamen TSPO V_T_ (eFigure in Supplement 1).

## Discussion

This case-control study is, to our knowledge, the first study to assess brain gliosis in postacute sequelae of SARS-CoV-2 infection. We focused our sample to represent the common clinical phenomenon of depressive or cognitive symptoms after acute mild or moderate SARS-CoV-2 (COVID-DC). We found generalized differences in TSPO V_T_ between persons with COVID-DC and healthy control participants, most prominent in the ventral striatum and dorsal putamen, and that greater severity of motor slowing correlated with higher dorsal putamen TSPO V_T_. These findings have important implications for understanding the pathology of COVID-DC and for developing clinical interventions.

Evaluations of TSPO in the brain of inflammatory states and neuropsychiatric disease indicate that the best explanation for greater TSPO V_T_ is greater density of activated microglia, and to a lesser extent, activated astroglia.^14^ This interpretation may be further nuanced to include some microglia and astroglia that may be transitioning toward an activated state and that a low level of TSPO may occur in other cells, such as endothelial cells. Among the regions selected for comparison between groups, the ventral striatum and dorsal putamen were most different; thus, these regions were prioritized in the interpretations. The largest differences in the ventral striatum and dorsal putamen regions also argue for these regions having the greatest injury. In disease with gliosis, the regions most affected typically have the most elevated TSPO V_T_, such as the hippocampus in Alzheimer disease,^17,29^ the cortico-striatal-thalamic circuit involving orbitofrontal cortex in obsessive compulsive disorder,^30^ or the focal injury of a stroke.^31^ Elevations in TSPO V_T_ in other brain regions, such as the anterior cingulate cortex and hippocampus included in the initial grouping of regions compared, likely represent a lower level of gliosis (compared with the ventral striatum and dorsal putamen). Plausible explanations to account for this lower gliosis level include concurrent injury from a common cause, spread of gliosis from the striatum through paracrine effects of proinflammatory cytokines, or striatal injury releasing damage-associated molecular patterns, of which the latter 2 may stimulate more gliosis beyond the striatum.^9^

**Figure 3.**
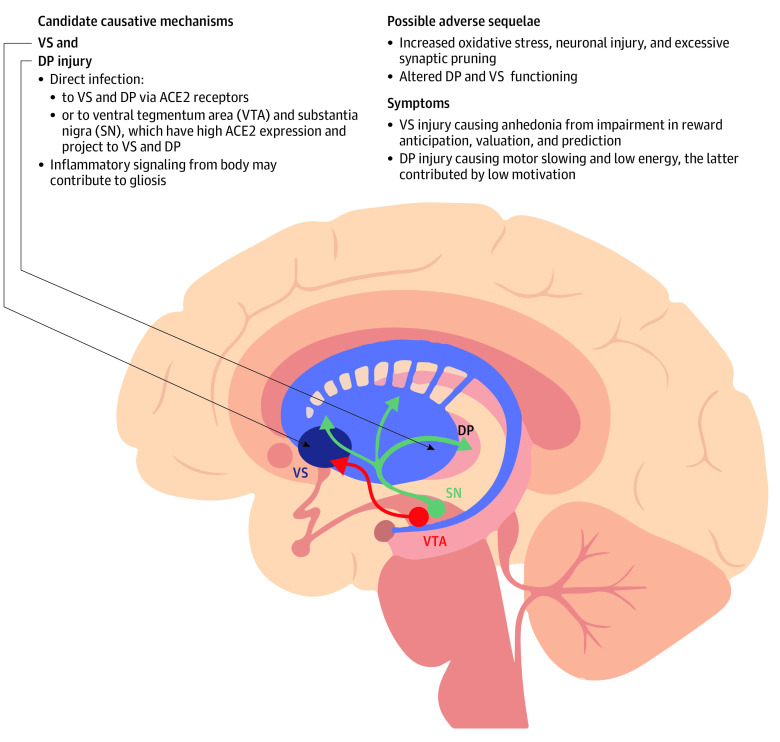
Theoretical Implications of Dorsal Putamen (DP) And Ventral Striatum (VS) Gliosis ACE2 indicates angiotensin-converting enzyme 2.

Injury to the ventral striatum and dorsal putamen is a plausible explanation for evidence of gliosis and is consistent with many symptoms observed in COVID-DC. Aberrant ventral striatum function may lead to anhedonia,^18,19^ and dorsal putamen injury is associated with motor slowing and low motivation or energy, which are also prominent symptoms of COVID-DC.^18^ A plausible interpretation of the negative correlation between finger-tapping speed and ventral striatum TSPO V_T_ is that the degree of injury from SARS-CoV-2 was associated with both poorer ventral striatum function and greater TSPO V_T_, leading to a correlation between the 2 measures. Possible mechanisms leading to gliosis in striatum include a combination of region-specific and nonspecific influences,^10^ such as signaling to the brain from elevated bodily inflammation, direct viral injury to the striatum, or viral injury to the substantia nigra or ventral tegmental area cells projecting to the striatum.^32,33^ The latter mechanism is consistent with reports of greater angiotensin-converting enzyme 2 messenger RNA in the substantia nigra^32^ and ventral tegmental area^33^ relative to other regions (Figure 3). Once gliosis is established, it may also self-perpetuate through multiple mechanisms, such as paracrine signaling or eliciting damage-associated molecular patterns from local injury through release of reactive molecule species, further stimulating gliosis.^9^ In summary, our favored explanation of the findings is a combination of direct virally induced injury to the striatum and projections to striatum, with additional general brainwide effects of virally induced injury and elevated bodily inflammatory signaling–initiated brain gliosis. The gliosis is maintained through paracrine effects, autocrine effects, and gliosis-induced neuronal injury that releases damage-associated molecular patterns. The injury and gliosis are mainly centered in the dorsal putamen and ventral striatum; thus, functions critical to these structures, such as hedonic responses, maintenance of motor speed, and motivation, are affected (Figure 3).

A key clinical implication of our findings is that addressing adverse effects of gliosis in COVID-DC may have therapeutic implications. Gliosis, which may include greater density of activated microglia and astroglia, is a response to injury that may have curative elements, such as removing cellular debris and producing neurotrophic factors, as well as damaging elements, such as creating reactive oxygen species and excessive synaptic removal. The damaging elements of chronic gliosis have been implicated in the persistence and progression of symptoms in neuropsychiatric disease (eDiscussion in Supplement 1).^9^ Hence, clinical trials of novel interventions to COVID-DC may consider suppressing adverse consequences of gliosis or suppressing gliosis entirely. While there is evidence for a modest effect of serotonin reuptake inhibitor antidepressants on reducing TSPO V_T_ in some circumstances,^27,34^ there are several medications in phase 2 or phase 3 trials in humans deliberately intentioned to target gliosis that could be studied in COVID-DC. For example, some TSPO binding medications have been associated with reduction in oxidative stress and synaptic loss during gliosis in rodents^35^; and P2X7 inhibitors reduce proliferation of microglia.^36^ However, it is acknowledged that the present study does not prove causality of symptoms from gliosis and that the influence of gliosis would ultimately be best determined in humans by the outcome of clinical interventions targeting gliosis. Moreover, it is possible that neuronal injury initially caused the gliosis and led to symptoms and that the ongoing gliosis plays only a modest role in perpetuating symptoms or further injury.

### Limitations

This study has several limitations. First, there may be heterogenous mechanisms for depressive and cognitive symptoms after COVID-19 illness, and our data represent cases after mild to moderate COVID-19 illness, a common clinical situation associated with approximately 95% of recent cases.^2^ Study outcomes may differ after severe COVID-19 infection with hospitalization, which may involve mechanisms of hypoxia and vascular occlusion (eDiscussion in Supplement 1). For example, a preprint report of 2 such cases scanned with [^18^F]DPA-714 (or [^18^F]-labeled *N,N*-diethyl-2-[4-(2-fluoroethoxy)phenyl]-5,7-dimethylpyrazolo[1,5-a]pyrimidine-3-acetamide) PET finds regional elevations in an index of TSPO binding (TSPO BP_ND_) greater than 70%, suggestive of larger magnitude elevations in TSPO level.^37^ Second, characterization of postacute COVID-19 symptoms is recent and ongoing; thus, some prominent symptoms observed in the present study, such as anhedonia and low motivation, were identified in the literature after the symptom measures for this study were chosen. It would be advantageous to include more targeted assessments for anhedonia and motivation in future imaging studies of COVID-DC. Third, elevated TSPO expression is not completely specific to glial cells. Although most TSPO in neuropsychiatric disease is typically expressed in microglia, and to a lesser extent in astroglia, the next most common cellular expression is in endothelial cells.^14,35^ However, endothelial cell content is unlikely to fully account for the findings since the spatial extent of endothelial cells is limited to surrounding blood vessels,^38^ which is less than the vascular contribution to PET, and the vascular contribution to PET is less than 5% of the input signal in the brain.^39^ Fourth, this study was cross-sectional; thus, the duration of persistently elevated TSPO V_T_ is not yet known. However, given the lack of correlation between time since acute COVID-19 illness and TSPO V_T_, persistent elevations in TSPO V_T_ are likely in individuals who, as in this sample, have persistent symptoms of COVID-DC. Finally, while our main findings of TSPO V_T_ associating with illness state and a correlation of the dorsal striatal TSPO V_T_ with finger-tapping test performance reflect important associations between brain changes and clinical symptoms, they do not prove that elevations in TSPO V_T_ (or gliosis) cause the symptoms of the disease state or cause poorer performance on the finger-tapping test.

## Conclusions

In this case-control study, evidence of elevated gliosis in the brain was found in patients with COVID-DC, most prominently in the ventral striatum and dorsal putamen. Also, symptoms of motor retardation, anhedonia, and low motivation leading to low energy were frequently present, collectively raising the possibility of injury to the ventral striatum and dorsal putamen. Consistent with the possibility of ongoing injury in the dorsal putamen, greater TSPO V_T_ was correlated with slower performance on the finger-tapping test in COVID-DC cases. Therapeutics to reduce gliosis, or more selectively the harmful effects of gliosis, may be helpful for COVID-DC, and gliosis-targeting treatments, such as TSPO and P2X7-binding medications, should be studied. However, it is also possible that the gliosis is a response to neuronal injury and has a limited role in maintaining symptoms and that preventative neuroprotective interventions would ultimately be more clinically impactful.

## Notes

Supplement 1.
eMethods

eResults

eDiscussion
**eFigure.** TSPO V_T_ Plotted in Relation to Time of Acute COVID-19 **eTable 1.** Relationship of Regional TSPO V_T_ With Neurocognitive Test Scores in COVID-DC **eTable 2.** Difference in Ventral Striatum and Dorsal Putamen TSPO V_T_ Between Groups Relative to TSPO V_T_ in Other Brain Regions

Supplement 2.
Data Sharing Statement
