# Supplementary material for: Neuroinflammation After COVID-19 With Persistent Depressive and Cognitive Symptoms
Source: JAMA Psychiatry. 2023 May 31;80(8):787–95. doi: 10.1001/jamapsychiatry.2023.1321 (PMC10233457; doi:10.1001/jamapsychiatry.2023.1321)
Supplement: Supplement 1. — eMethods eResults eDiscussion eFigure. TSPO VT Plotted in Relation to Time of Acute COVID-19 eTable 1. Relationship of Regional TSPO VT With Neurocognitive Test Scores in COVID-DC eTable 2. Difference in Ventral Striatum and Dorsal Putamen TSPO VT Between Groups Relative to TSPO VT in Other Brain Regions [file jamapsychiatry-e231321-s001.pdf]

Supplementary Online Content

Braga J, Lepra M, Kish SJ, et al. Neuroinflammation after COVID-19 with persistent depressive and cognitive symptoms. Published online May 31, 2023. *JAMA Psychiatry*.
doi:10.1001/jamapsychiatry.2023.1321

eMethods.

eResults.

eDiscussion.

eReferences.

eFigure. TSPO V_T Plotted in Relation to Time of Acute COVID-19.

eTable 1. Relationship of Regional TSPO V_T With Neurocognitive Test Scores in COVID-DC

eTable 2. Difference in Ventral Striatum and Dorsal Putamen TSPO V_T Between Groups Relative to TSPO V_T in Other Brain Regions

This supplementary material has been provided by the authors to give readers additional information about their work.

© 2023 Braga J et al. *JAMA Psychiatry.*

eMETHODS

Participants

73 COVID-DC participants consented to participate in the study and completed screening procedures. 35 were eligible after initial screening, and among them, 25 participants decided to participate. Of these, the arterial line insertion was unable to be completed in three participants and there were arterial sampling pump failures in two participants. Hence 20 participants with COVID-DC completed [^{18}F]FEPPA positron emission tomography (PET) scanning. Recruitment of 20 healthy participants, completed prior to the COVID-19 pandemic, were described previously[1]. All COVID-DC cases had at least one previous documented acute episode of COVID-19 (n=19 one episode, n=1 two episodes). Most (n=17) previously had acute COVID-19 of moderate severity.

Inclusion/Exclusion Criteria

All participants met additional exclusionary enrollment criteria required for PET scanning with arterial blood sampling including: not pregnant, not breastfeeding, no blood coagulation disorders, no ongoing anticoagulant use, no claustrophobia, no history of fainting due to blood withdrawals, size/weight not exceeding capacity of the PET and magnetic resonance imaging (MRI) scanners (approximately 160kgs). No presence of metal objects or implanted electrical devices in the body that would preclude MRI scanning was also required.

Previous acute COVID-19 was required to be of mild or moderate severity as defined by NIH and World Health Organization. Moderate COVID-19 is presence of clinical symptoms of pneumonia but not severe enough to require ongoing use of supplementary oxygen; and mild COVID-19 is symptoms but no evidence of pneumonia or hypoxia. Participants were asked a list of questions that included whether they had individual symptoms of pneumonia as well as whether they received supplemental oxygen.

PET and MRI Scanning

[^{18}F]FEPPA was of high radiochemical purity with a minimum threshold of 95% and high specific activity (mean±SD, 196±140 terabecquerels/mmol at the time of injection). There was no significant difference in specific activity between groups. A transmission scan measured using a single photon point source, ^{137}Cs ($t_{1/2}$=30·2 years, Eγ=662 keV) was acquired immediately before the acquisition of the emission scan. For the emission scan, an intravenous bolus injection of [^{18}F]FEPPA was given and data was acquired for 125 minutes after (34 time frames: 1 frame of variable length, 5 × 30 s, 1 × 45 s, 2 × 60 s, 1 × 90 s, 1 × 120 s, 1 × 210 s, and 22 × 300 s). Attenuation correction was done using a ^{137}Cs transmission scan acquired in 64 bit list mode, which is converted into a 511 keV attenuation correction image.[2] Emission images were acquired in 64 bit list mode, and were later reconstructed from 3D sinograms. Key steps in reconstruction include accounting for the octagonal design of the tomograph,[3] correction for photon attenuation, detector normalization, and scatter in the 3D sinograms,[4] fourier rebinning to convert 3D into 2D sinograms,[5] reconstruction into image space using a 2D filtered back projection algorithm with a HANN filter at Nyquist cut-off frequency and calibration of the images to nCi/cc.

© 2023 Braga J et al. *JAMA Psychiatry.*

Arterial blood was collected for the first 22·5 min after radiotracer injection at a rate of 2·5 mL/min and blood radioactivity levels were measured using an automatic blood sampling system (Model PBS-101, Veenstra Instruments, Joure, Netherlands). Additionally, 7 mL blood samples were drawn manually at -5, 2·5, 7, 12, 15, 20, 30, 45, 60, 90, and 120 min following tracer injection. The delay, dispersion and metabolite corrected input function were created as previously described[6].

Magnetic resonance imaging (MRI) scans were acquired at the CAMH Brain Health Imaging Centre on a GE Discovery MR750 3T scanner (General Electric Medical Systems, Milwaukee, WI) equipped with an 8-channel headcoil. T_1-weighted MRI acquired in 3D of slice thickness 0·9mm were obtained (TR 6·7ms, TE 3·0ms, flip angle 8°, NEX=1, acquisition matrix 256 x 256; FOV 23cm, duration 4 min 42 s). Regions of Interest were delineated using in-house image analysis software ROMI.[7] In brief, a standard brain template (International Consortium for Brain Mapping/Montreal Neurological Institute 152 MRI) containing a set of predefined cortical and subcortical ROIs (based upon the neuroanatomy atlas of Duvernoy[8] but consistent with the atlas of Talairach[9]; the division of the striatum is from Mawlawi et al.[10] and the prefrontal cortex subregions are derived from the cytoarchitectural definitions of Rajkowska).[11-13] These ROI are transformed and deformed (SPM normalization; Wellcome Dept. of Cognitive Neurology, London, UK; http://www.fil.ion.ucl.ac.uk/spm/) to fit each individual high-resolution T1 MRI. Each individual's set of automatically created ROIs is then refined by iteratively including and deleting voxels based on the probability of each voxel belonging to grey matter (SPM8 segmentation, Wellcome Department of Cognitive Neurology, London, UK; http://www.fil.ion.ucl.ac.uk/spm). Each MRI is co-registered to the summed PET image using normalized mutual information algorithm implemented under SPM8. [8] The resulting transformation is applied to the individual's refined ROIs and then resliced to match the dimensions of the PET images. The location of the ROI is verified by visual assessment of the ROI on the coregistered MRI and summated [^{18}F]FEPPA PET image. Time activity curves are visually inspected and if evidence for motion artifact is present a motion correction algorithm is applied. Head movement in the dynamic PET acquisition is corrected using frame-by-frame realignment. A normalized mutual information algorithm is applied with SPM8 (Wellcome Trust Centre for Neuroimaging, London, UK) to coregister each frame to the 12th frame after the radioligand arrives to the field of view, which shows a high signal-to-noise ratio. Parameters from the normalized mutual information are then applied to the corresponding attenuation-corrected dynamic images to generate a movement-corrected dynamic image.

The two tissue compartment analysis, previously validated for this radiotracer[6], was performed using PMOD Kinetic Modeling Tool (PKIN) version 4·2 (PMOD technologies, Zurich, Switzerland) to measure translocator total distribution volume (TSPO V_T). All statistical analyses were conducted using IBM SPSS Statistics for Windows version 25·0 (IBM Corp., Armonk, N.Y., USA.)

Selection of ROIs

Symptoms of anhedonia, motor retardation and low energy (one component of which is low motivation[14]) are prominent in COVID-DC[15-17]. It is well established that the ventral striatum participates in anticipating, predicting, valuing reward[18-20]. The dorsal putamen participates in maintaining normal movement speed and motivation[20-23]. Human diseases with injury to ventral

© 2023 Braga J et al. *JAMA Psychiatry.*

striatum are associated with anhedonia such as in PD[24-26], and 1-methyl-4-phenyl-1,2,3,6-tetrahydropyridine (MPTP) toxicity[27-29]. Similarly, human diseases with injury in dorsal striatum are associated with motor retardation and amotivation such as in PD[24-26], progressive supranuclear palsy[30,31], multiple system atrophy[30-32] and MPTP toxicity[27-29]. It is well established that tests of motor speed like the Finger Tapping Test are impaired in such illnesses.

The PFC and ACC were chosen because of their role in mood regulation circuitry and affect dysregulation of MDD[33]. Depressed mood induction is known to influence the activity of these structures[33] and gliosis in these structures is associated with major depressive episodes[34]. A commonly used measure for overall severity of major depressive episodes is the Hamilton Depression Rating Scale so this was selected as a correlate to evaluate. Hippocampal function is critical to consolidation of declarative memories and verbal memory[35,36]. Moreover, the magnitude of injury to hippocampus in neurodegenerative diseases is associated with severity of impaired cognitive functioning[35,36]. Since hippocampus function participates in a number of processes for the present study and at the design phase of the present study cognitive concerns after COVID-19 were still largely under investigation, we prioritized patient self report of symptoms via the Cognitive Failures Questionnaire, but as an exploration also conducted a group of neuropsychological tests, some of which require intact hippocampus functioning (Supplemental Table 1).

Neuropsychological Assessment

Neuropsychological testing included a broader assessment of different domains including processing speed; visuospatial functioning; learning and memory; attention and executive function; and psychological testing assessed a wide range of depressive and anxiety related symptoms (Supplemental Table 1). The Finger Tapping Test[37,38], a well-established measure of motor speed is prioritized because it is demonstrably affected in injury to the dorsal putamen; a structure that participates in voluntary movement control[39]. The relationship between additional cognitive tests and TSPO V_T within regions implicated in the performance of the cognitive test were also assessed. Tests related to processing speed and psychomotor functioning included the Digit Symbol coding subtest of the Wechsler Adult Intelligence Scale-III[37], Word and Color trials on the Stroop Color and Word Test, and Comprehensive Trail Making Test Trails 1 to 3[40]. Other measures and their respective domains included: CTMT Trails 4 & 5[40] and Color-Word trial on the Stroop Test, executive function; Hopkins Verbal Learning Test-Revised (HVLT-R)[41] and Brief Visuospatial Memory Test-Revised[42], verbal and visual learning and memory; and Benton Judgement of Line Orientation (JLO)[43], visuospatial functioning. Outcomes for test performance, decided *a priori*, were T scores reflecting individual performance relative to normative peer samples, derived from published demographically stratified or adjusted data: CTMT, HVLT-R, Stroop Test, and BVMT-R according to participant age, sex, and/or education; Finger Tapping Test[37,44] and JLO[45] to participant age and sex; and the Digit Symbol coding subtest to participant age only (Supplemental Table 1).

Power

Detection of a 20% difference, given the standard deviation of TSPO V_T in healthy, corresponds to an effect size of approximately 0.7. For a repeated measures analysis of variance to detect

© 2023 Braga J et al. *JAMA Psychiatry.*

between group effects, applying a f value of 0.35, evaluation of TSPO V_T in five regions correlated with r=0·45 corresponds to a power of 80% in a comparison of 20 versus 20 participants.[46]

Multiple Comparisons

For the main analysis, the group effect across the five regions (ventral striatum, dorsal putamen, prefrontal cortex, anterior cingulate cortex and hippocampus) was considered significant at a threshold of 0.05. For the correlations with regional TSPO V_T, there were 3 main correlates assessed: motor speed with the Finger Tapping Test, MDE severity with Hamilton Depression Rating Scale, and Cognitive Failures Questionnaire. However, the Finger Tapping Test correlated highly and negatively with the Hamilton Depression Rating Scale score (r=-0.70, p<0.001). So there were two independent correlates and the threshold was adjusted to 0.025.

Voxel Based Analysis

In an exploratory analysis, the logan model was used to measure TSPO V_T at the voxel level for each participant applying ROMI software[47]. Consistent with previous use of this approach for [^{18}F]FEPPA[48], the TSPO V_T results at the voxel level were highly correlated with TSPO V_T found with the logan model applied to time activity curves from the same regions of interest across participants, exemplified in the ventral striatum and dorsal putamen (r=0.95 and 0.97, respectively). Also consistent with previous use of this approach for [^{18}F]FEPPA, the regional TSPO V_T found with the logan model correlated highly with the TSPO V_T measured applying the two tissue compartment model results across participants as exemplified in the same regions (r=0.91 and 0.97, respectively).

In SPM12, individual participant PD MRI were coregistered to the images composed of the mean summed counts of each [^{18}F]FEPPA image with a mutual information algorithm, the latter which is in the same space as the voxel based V_T images. Then the PD MRI were spatially normalized to the 152 MNI template in SPM12, applying the default options with the same transformations and deformations applied to the voxel TSPO V_T PET images. Then the voxel TSPO V_T images, now in MNI space, were smoothed with the default option. Finally, an ANOVA was applied evaluating effect of group, after applying a factor of genotype MAB versus HAB.

eRESULTS

MRI Evaluation

MRI were clinically unremarkable in the COVID group except for 7 cases with mucous retention cysts in maxillary sinus, sphenoid sinuses or ethmoid air cells which was also associated with mucosal thickening in 4 of these 7 cases.

Statistical Analyses

Post-hoc analysis showed that differences between participants and controls in VS or DP TSPO V_T relative to other regions in the group analysis, in which TSPO V_T in the other regions was

© 2023 Braga J et al. *JAMA Psychiatry.*

applied as a covariate, were mostly significant or if not, at trend level (Supplemental Table 2). TSPO V_T in VS and DP were highly correlated in COVID-DC participants with a correlation coefficient (r) of 0.91 ($p<0.001$). History of past MDE was not correlated with TSPO V_T in any region assayed.

Relationship of Other Neurological Symptoms to TSPO V_T

Presence of olfactory symptoms was not associated with orbitofrontal cortex TSPO V_T adjusted for genotype difference (ANOVA, mean difference, 0.8; 95% CI, -1.9 to 3.4; p=0.5). Presence of impaired taste was not associated with insular cortex TSPO V_T adjusted for genotype difference (ANOVA, mean difference, 1.7; 95% CI, -1.1 to 4.5, p=0.2).

Voxel Based Analysis

In the comparison of patients greater than controls in SPM12, no peaks were found to be significant after correcting for family wise error or false discovery rate. At the uncorrected threshold of p=0.05, a cluster inclusive of essentially all grey matter regions of the brain was present (k=146981 voxels) which was significant after applying family wise error ($p<0.001$). In the comparison of controls greater than patients, no voxels were significant at the corrected threshold of p=0.05.

eDISCUSSION

In the present study we found no relationship between TSPO V_T and duration of illness post-acute COVID-19. While the data is cross-sectional, it suggests that in COVID-DC cases with persistent symptoms of MDE, TSPO V_T, an index of gliosis, does not decline over months. In a previous study of MDE secondary to MDD prior to COVID-19, it was found in cross-sectional data that long durations of untreated MDE, that is, greater than 10 years, were associated with greater TSPO V_T across most grey matter regions[1]. This was interpreted as likely reflecting progressive gliosis in those with chronic MDE of MDD. Since COVID-19 is a relatively recent event, it is not known whether similarly long durations of COVID-DC beyond 10 years might be similarly associated with greater TSPO V_T.

An asset of the study design was that participants did not have a current MDE at the time of acute COVID-19. This was to avoid confounding of elevated TSPO V_T predating the episode of acute COVID-19. There are several lines of evidence to support that presence of a MDE, rather than a history of MDE is associated with elevated TSPO V_T. In a [^{18}F]FEPPA PET study, Li et al. demonstrated that reductions of symptoms with cognitive behavioural therapy resulted in normalization of TSPO V_T across brain regions assessed[49]. Hannestad et al. compared cases of major depressive disorder, most of whom did not meet threshold criteria for a major depressive episode, and found no difference in TSPO V_T in the regions assessed[50]. In addition, in antidepressant free MDE cases of MDD, Setiawan et al. found positive correlations between severity of MDE and TSPO V_T in most brain regions assessed such as the ACC, PFC and insula[1].

In one of the largest post mortem studies of gliosis after COVID-19, Matschke et al. reported prominent microglial activation in the brainstem whereas the present study did not find a prominent elevation of TSPO V_T in the midbrain[51]. Since, in contrast to the present study, the sample of Matschke et al. died of COVID-19, one could speculate that prominent gliosis in the brainstem, which controls key life supporting processes like respiration, might contribute to the

© 2023 Braga J et al. *JAMA Psychiatry.*

lethality of COVID-19. Brainstem gliosis has been implicated in other causes of death such as sudden infant death syndrome[52].

The voxel based analysis found generally greater TSPO V_T in patients than controls, a finding reasonably similar to region of interest results. No peak changes with a different anatomical definition, as compared to the ROI, were identified.

© 2023 Braga J et al. *JAMA Psychiatry.*

eREFERENCES

1. Setiawan E, Attwells S, Wilson AA, et al. Association of translocator protein total distribution volume with duration of untreated major depressive disorder: a cross-sectional study. *Lancet Psychiatry.* Apr 2018;5(4):339-347. doi:10.1016/S2215-0366(18)30048-8

2. Knoess C, Rist J, Michel C, et al. Evaluation of Single Photon Transmission for the HRRT. *IEEE Nucl Sci Symp.* 2003;3:1936-1940.

3. Karp J, Muehllehner G, Lewitt R. Constrained Fourier Space Method for Compensation of Missing Data in Emission Computed Tomography. *IEEE Transactions on Medical Imaging.* 1988;7(1).

4. Watson C, Newport D, Casey M, deKemp R, Beanlands R, Schmand M. Evaluation of simulation-base scatter correction for 3D PET cardiac imaging. *IEEE Transactions on Nuclear Science.* 1996;44:90-97.

5. Defrise M, Kinahan P, Townsend D, Michel C, Sibomana M, Newport D. Exact and Approximate Rebinning Algorithms for 3-D PET Data. *IEEE Transactions on Medical Imaging.* 1997;16(2):145-158.

6. Rusjan PM WA, Bloomfield PM, Vitcu I, Meyer JH, Houle S, et al. Quantitation of translocator protein binding in human brain with the novel radioligand [18F]-FEPPA and positron emission tomography. . *J Cereb Blood Flow Metab* 2011;31:1807-1816.

7. Rusjan P, Mamo D, Ginovart N, et al. An automated method for the extraction of regional data from PET images. *Psychiatry Res.* Jun 30 2006;147(1):79-89.

8. Duvernoy H. *The Human Brain: Surface, Blood Supply and Three Dimensional Sectional Anatomy.* New York: SpringerWien; 1999.

© 2023 Braga J et al. *JAMA Psychiatry.*

9. Talairach J, Tournoux P. *Co-Planar Stereotaxic Atlas of the Human Brain.* New York: Thieme Medical; 1988.

10. Mawlawi O, Martinez D, Slifstein M, et al. Imaging human mesolimbic dopamine transmission with positron emission tomography: I. Accuracy and precision of D(2) receptor parameter measurements in ventral striatum. *J Cereb Blood Flow Metab.* Sep 2001;21(9):1034-1057.

11. Rajkowska G, Goldman-Rakic PS. Cytoarchitectonic definition of prefrontal areas in the normal human cortex: I. Remapping of areas 9 and 46 using quantitative criteria. *Cereb Cortex.* Jul-Aug 1995;5(4):307-322.

12. Rajkowska G, Goldman-Rakic PS. Cytoarchitectonic definition of prefrontal areas in the normal human cortex: II. Variability in locations of areas 9 and 46 and relationship to the Talairach Coordinate System. *Cereb Cortex.* Jul-Aug 1995;5(4):323-337.

13. Uylings HB, Sanz-Arigita EJ, de Vos K, Pool CW, Evers P, Rajkowska G. 3-D cytoarchitectonic parcellation of human orbitofrontal cortex correlation with postmortem MRI. *Psychiatry Res.* Jul 30 2010;183(1):1-20. doi:S0925-4927(10)00148-4 [pii]

10.1016/j.pscychresns.2010.04.012

14. National_Health_Service(UK). Long COVID: Fatigue. https://www.nhsinform.scot/long-term-effects-of-covid-19-long-covid/signs-and-symptoms/long-covid-fatigue. Published 2022. Accessed August 1, 2022.

15. Tavares-Junior JWL, de Souza ACC, Borges JWP, et al. COVID-19 associated cognitive impairment: A systematic review. *Cortex.* Jul 2022;152:77-97. doi:10.1016/j.cortex.2022.04.006

16. Lopez-Leon S, Wegman-Ostrosky T, Perelman C, et al. More than 50 long-term effects of COVID-19: a systematic review and meta-analysis. *Scientific reports.* 2021;11(1):1-12.

© 2023 Braga J et al. *JAMA Psychiatry.*

17. Lamontagne SJ, Winters MF, Pizzagalli DA, Olmstead MC. Post-acute sequelae of COVID-19: Evidence of mood & cognitive impairment. *Brain, Behavior, & Immunity-Health.* 2021;17:100347.

18. Schultz W. Reward functions of the basal ganglia. *Journal of neural transmission.* 2016;123(7):679-693.

19. Daniel R, Pollmann S. A universal role of the ventral striatum in reward-based learning: evidence from human studies. *Neurobiology of learning and memory.* 2014;114:90-100.

20. Haber S. Neuroanatomy of Reward: A View from the Ventral Striatum. In: Gottfried J, ed. *Neurobiology of Sensation and Reward.* Boca Raton (FL): CRC Press/Taylor and Francis; 2011.

21. Ghandili M, Munakomi S. Neuroanatomy, Putamen. In: *StatPearls.* Treasure Island (FL)2022.

22. Liljeholm M, O'Doherty JP. Contributions of the striatum to learning, motivation, and performance: an associative account. *Trends Cogn Sci.* Sep 2012;16(9):467-475. doi:10.1016/j.tics.2012.07.007

23. McGuigan S, Zhou SH, Brosnan MB, Thyagarajan D, Bellgrove MA, Chong TT. Dopamine restores cognitive motivation in Parkinson's disease. *Brain.* Mar 1 2019;142(3):719-732. doi:10.1093/brain/awy341

24. Armstrong MJ, Okun MS. Diagnosis and Treatment of Parkinson Disease: A Review. *JAMA.* Feb 11 2020;323(6):548-560. doi:10.1001/jama.2019.22360

25. Loas G, Krystkowiak P, Godefroy O. Anhedonia in Parkinson's disease: an overview. *J Neuropsychiatry Clin Neurosci.* Fall 2012;24(4):444-451. doi:10.1176/appi.neuropsych.11110332

26. Alberico SL, Cassell MD, Narayanan NS. The Vulnerable Ventral Tegmental Area in Parkinson's Disease. *Basal Ganglia.* Aug 1 2015;5(2-3):51-55. doi:10.1016/j.baga.2015.06.001

© 2023 Braga J et al. *JAMA Psychiatry.*

27. Snow BJ, Vingerhoets FJ, Langston JW, Tetrud JW, Sossi V, Calne DB. Pattern of dopaminergic loss in the striatum of humans with MPTP induced parkinsonism. *J Neurol Neurosurg Psychiatry.* Mar 2000;68(3):313-316. doi:10.1136/jnnp.68.3.313

28. Langston JW, Ballard P, Tetrud JW, Irwin I. Chronic Parkinsonism in humans due to a product of meperidine-analog synthesis. *Science.* Feb 25 1983;219(4587):979-980. doi:10.1126/science.6823561

29. Brown CA, Campbell MC, Karimi M, et al. Dopamine pathway loss in nucleus accumbens and ventral tegmental area predicts apathetic behavior in MPTP-lesioned monkeys. *Exp Neurol.* Jul 2012;236(1):190-197. doi:10.1016/j.expneurol.2012.04.025

30. Fabbrini G, Fabbrini A, Suppa A. Progressive supranuclear palsy, multiple system atrophy and corticobasal degeneration. In: Reus V, Lindqvist D, eds. *Handbook of Clinical Neurology.* Vol 165. Elsevier; 2019:155-177.

31. Kaasinen V, Kankare T, Joutsa J, Vahlberg T. Presynaptic Striatal Dopaminergic Function in Atypical Parkinsonism: A Metaanalysis of Imaging Studies. *J Nucl Med.* Dec 2019;60(12):1757-1763. doi:10.2967/jnumed.119.227140

32. Gilman S. Biochemical changes in multiple system atrophy detected with positron emission tomography. *Parkinsonism Relat Disord.* Jul 2001;7(3):253-256. doi:10.1016/s1353-8020(00)00066-3

33. Ressler KJ, Mayberg HS. Targeting abnormal neural circuits in mood and anxiety disorders: from the laboratory to the clinic. *Nat Neurosci.* Aug 28 2007;10(9):1116-1124.

34. Meyer JH, Cervenka S, Kim M-J, Kreisl WC, Henter ID, Innis RB. Neuroinflammation in Psychiatric Disorders: PET Imaging and Promising New Targets. *Lancet Psychiatry.* 2020;7(12):1064-1074.

© 2023 Braga J et al. *JAMA Psychiatry.*

35. Bonner-Jackson A, Mahmoud S, Miller J, Banks SJ. Verbal and non-verbal memory and hippocampal volumes in a memory clinic population. *Alzheimers Res Ther.* Oct 15 2015;7(1):61. doi:10.1186/s13195-015-0147-9

36. Bettio LEB, Rajendran L, Gil-Mohapel J. The effects of aging in the hippocampus and cognitive decline. *Neurosci Biobehav Rev.* Aug 2017;79:66-86. doi:10.1016/j.neubiorev.2017.04.030

37. Spreen O, Strauss E. *A Compendium of Neuropsychological Tests: Administration, Norms, Commentary.* second edition ed. New York: Oxford University Press; 1998.

38. Reitan RM, Wolfson D. *The Halstead-Reitan neuropsychological test battery: Theory and clinical interpretation.* Vol 4: Reitan Neuropsychology; 1985.

39. Graybiel AM, Aosaki T, Flaherty AW, Kimura M. The basal ganglia and adaptive motor control. *Science.* Sep 23 1994;265(5180):1826-1831.

40. Reynolds C. *CTMT-2: Comprehensive Trail-Making Test Second Edition-Complete Kit.* PAR; 2020.

41. Brandt J, Benedict, R. H. B. *Hopkins Verbal Learning Test-Revised TM. Professional Manual.* . Lutz, FL: PAR; 2001.

42. Benedict RHB. *BVMT-R Recognition Stimulus Booklet. Forms 1 through 6.* . Lutz, FL: PAR; 1997.

43. Benton AL, Sivan, A. B., Hamsher, K. deS., Varney, N.R., Spreen. O. *Judgement of Line Orientation* Lutz, FL: PAR; 1983.

44. Heaton RK. *Revised comprehensive norms for an expanded Halstead-Reitan Battery: Demographically adjusted neuropsychological norms for African American and Caucasian adults, professional manual.* Psychological Assessment Resources; 2004.

45. Konen T, Karbach J. Self-Reported Cognitive Failures in Everyday Life: A Closer Look at Their Relation to Personality and Cognitive Performance. *Assessment.* Jul 1 2018:1073191118786800. doi:10.1177/1073191118786800

46. Faul F, Erdfelder E, Buchner A, Lang AG. Statistical power analyses using G*Power 3.1: tests for correlation and regression analyses. *Behav Res Methods.* Nov 2009;41(4):1149-1160. doi:41/4/1149 [pii]

10.3758/BRM.41.4.1149

47. Rusjan P, Mamo D, Ginovart N, et al. An automated method for the extraction of regional data from PET images. *Psychiatry Research: Neuroimaging.* 2006;147(1):79-89.

48. Hafizi S, Tseng H-H, Rao N, et al. Imaging microglial activation in untreated first-episode psychosis: a PET study with [18F] FEPPA. *American Journal of Psychiatry.* 2017;174(2):118-124.

49. Li H, Sagar AP, Kéri S. Translocator protein (18kDa TSPO) binding, a marker of microglia, is reduced in major depression during cognitive-behavioral therapy. *Progress in Neuro-Psychopharmacology and Biological Psychiatry.* 2018/04/20/ 2018;83:1-7. doi:https://doi.org/10.1016/j.pnpbp.2017.12.011

50. Hannestad J, DellaGioia N, Gallezot J-D, et al. The neuroinflammation marker translocator protein is not elevated in individuals with mild-to-moderate depression: A [11C]PBR28 PET study. *Brain, Behavior, and Immunity.* 2013/10/01/ 2013;33:131-138. doi:https://doi.org/10.1016/j.bbi.2013.06.010

51. Matschke J, Lütgehetmann M, Hagel C, et al. Neuropathology of patients with COVID-19 in Germany: a post-mortem case series. *The Lancet Neurology.* 2020/11/01/ 2020;19(11):919-929. doi:https://doi.org/10.1016/S1474-4422(20)30308-2

© 2023 Braga J et al. *JAMA Psychiatry.*

52. Takashima S, Armstrong D, Becker L, Bryan C. Cerebral hypoperfusion in the sudden infant death syndrome? brainstem gliosis and vasculature. *Annals of Neurology*. 1978;4(3):257-262. doi:https://doi.org/10.1002/ana.410040312

© 2023 Braga J et al. *JAMA Psychiatry.*

SUPPLEMENTAL FIGURE & TABLES

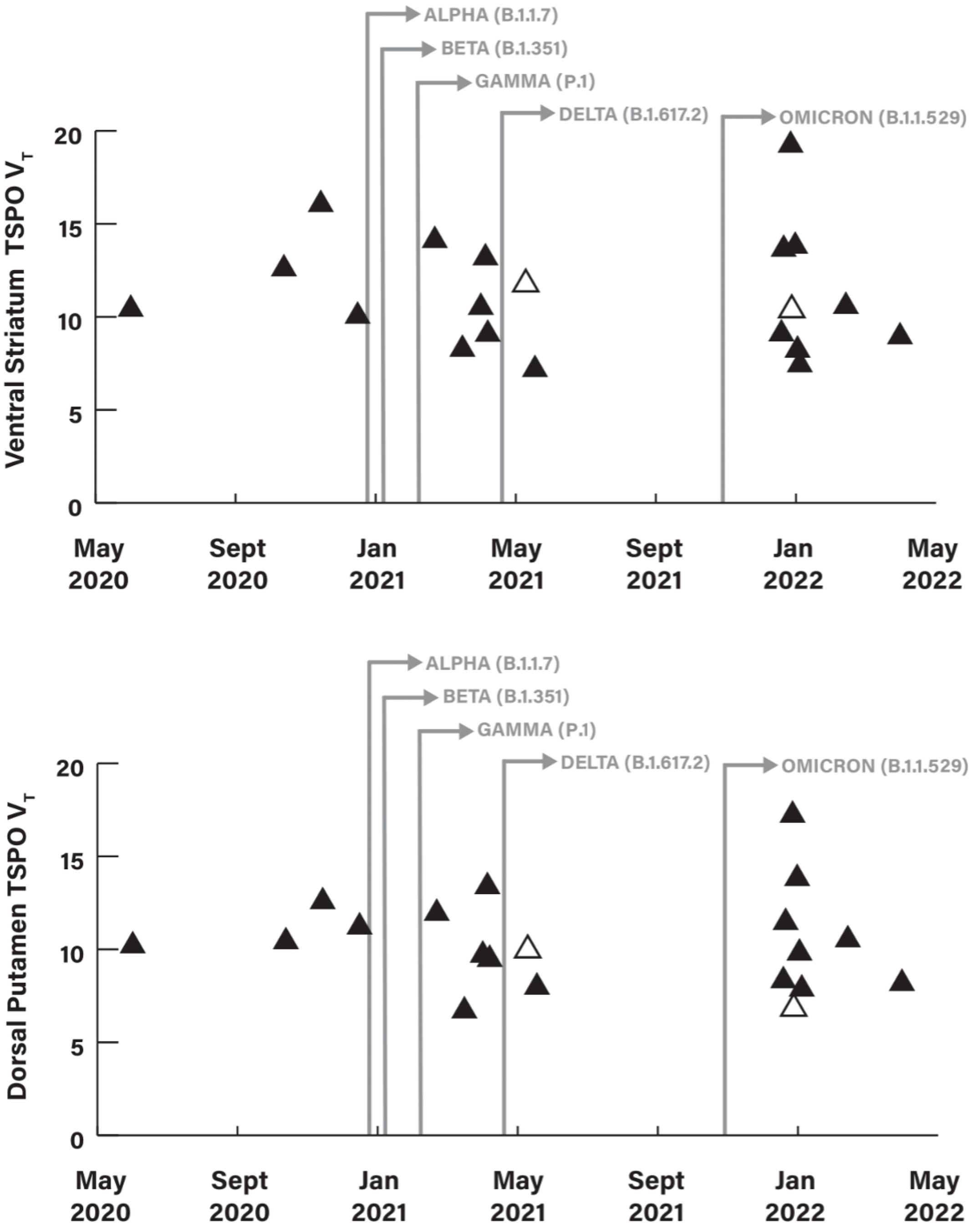

eFigure. TSPO V_T plotted in relation to time of acute COVID-19. Onset time of variants in Canada is concurrently labelled. TSPO V_T = Translocator protein total distribution volume. HAB ▲/MAB △ = High-affinity/Mixed-affinity binder based on rs6971 genotype.

© 2023 Braga J et al. *JAMA Psychiatry.*

eTable 1. Relationship of Regional TSPO V_T with Neurocognitive Test Scores in COVID-DC

	Region of Interest														
	Ventral Striatum			Dorsal Putamen			Hippocampus			Anterior Cingulate Cortex			Prefrontal Cortex		
	r	95% CI	*p*	r	95% CI	*p*	r	95% CI	*p*	r	95% CI	*p*	r	95% CI	*p*
**Psychomotor and Processing Speed**															
**Finger Tapping Test (DH)[a]**	- 0.30	-0.67 to 0.18	0.21	-0.53	-0.79 to -0.09	0.02	- 0.31	-0.67 to 0.17	0.20	- 0.50	-0.78 to -0.06	0.03	- 0.41	-0.73 to 0.05	0.08
**Finger Tapping Test (NDH)[a]**	- 0.34	-0.70 to 0.15	0.17	- 0.51	-0.79 to -0.06	0.03	- 0.34	-0.69 to 0.15	0.17	- 0.42	-0.74 to 0.06	0.08	- 0.39	-0.72 to 0.10	0.11
**CTMT Inhibitory Control Index[a]**	0.38	-0.09 to 0.71	0.11	0.20	-0.28 to 0.60	0.40	0.38	-0.09 to 0.71	0.11	0.18	-0.30 to 0.58	0.47	0.15	-0.32 to 0.57	0.53
**STROOP Word Trial[a]**	- 0.22	-0.61 to 0.26	0.37	- 0.35	-0.69 to 0.13	0.15	- 0.33	-0.68 to 0.15	0.17	- 0.23	-0.62 to 0.25	0.35	- 0.23	-0.62 to 0.25	0.35
**STROOP Colour Trial[a]**	- 0.07	-0.50 to 0.40	0.79	- 0.24	-0.63 to 0.24	0.31	- 0.14	-0.56 to 0.34	0.57	- 0.27	-0.64 to 0.22	0.27	- 0.29	-0.66 to 0.19	0.24
**WAIS-III Digit Symbol Coding Task[b]**	- 0.04	-0.49 to 0.42	0.86	-0.22	-0.62 to 0.26	0.36	-0.09	-0.52 to 0.38	0.72	-0.35	-0.69 to 0.13	0.15	-0.30	-0.66 to 0.18	0.22
**Executive Function**															
**CTMT Set-Shifting Index[a]**	0.11	-0.36 to 0.54	0.65	0.13	-0.35 to 0.55	0.60	0.18	-0.30 to 0.58	0.47	0.00	-0.45 to 0.46	0.99	-0.04	-0.48 to 0.43	0.89
**STROOP Colour-Word Trial[a]**	- 0.17	-0.58 to 0.31	0.49	- 0.19	-0.59 to 0.29	0.44	- 0.22	-0.62 to 0.26	0.36	- 0.33	-0.68 to 0.15	0.18	- 0.29	-0.66 to 0.19	0.23
**Learning and Memory**															
**HVLT-R Total Recall[a]**	- 0.13	-0.55 to 0.34	0.60	- 0.20	-0.60 to 0.28	0.41	0.05	-0.41 to 0.50	0.83	- 0.07	-0.51 to 0.40	0.77	- 0.22	-0.62 to 0.26	0.36
**HVLT-R Delayed Recall[a]**	- 0.10	-0.54 to 0.39	0.69	- 0.05	-0.51 to 0.43	0.84	0.07	-0.41 to 0.52	0.77	- 0.04	-0.50 to 0.44	0.88	- 0.26	-0.65 to 0.24	0.30
**HVLT-R Retention[a]**	- 0.04	-0.49 to 0.42	0.87	0.23	-0.25 to 0.62	0.34	0.21	-0.27 to 0.61	0.38	0.17	-0.30 to 0.58	0.48	0.02	-0.44 to 0.47	0.95
**BVMT-R Total Recall[a]**	0.35	-0.13 to 0.69	0.14	0.17	-0.31 to 0.58	0.48	0.37	-0.10 to 0.71	0.12	0.17	-0.31 to 0.58	0.50	0.20	-0.28 to 0.60	0.41
**BVMT-R Learning[a]**	- 0.50	-0.78 to -0.06	0.03	- 0.30	-0.66 to 0.18	0.21	- 0.33	-0.68 to 0.14	0.17	- 0.39	-0.72 to 0.08	0.10	- 0.44	-0.75 to 0.02	0.06
**BVMT-R Delayed Recall[a]**	0.19	-0.29 to 0.59	0.44	0.09	-0.38 to 0.52	0.72	0.28	-0.21 to 0.65	0.26	0.05	-0.41 to 0.49	0.84	0.08	-0.39 to 0.52	0.73
**Visual-Spatial**															
**Benton JLO[c]**	-0.06	-0.50 to 0.41	0.82	-0.15	-0.56 to 0.33	0.55	0.03	-0.43 to 0.48	0.91	-0.07	-0.51 to 0.39	0.76	-0.09	-0.52 to 0.38	0.71

Data are the correlation coefficients (r) between regional TSPO V_T values and neuropsychological test scores. TSPO V_T = Translocator protein total distribution volume. COVID-DC = COVID-19 and depression with/without other cognitive symptoms. DH = dominant hand. NDH = non-dominant hand. CTMT = Comprehensive Trail Making Test. WAIS = Wechsler Adult Intelligence Scale. HVLT-R = Hopkins Verbal Learning Test-Revised. BVMT-R = Brief Visuospatial Memory Test-Revised. JLO = Judgement of Line Orientation. TSPO V_T values of mixed-affinity binders are multiplied by 1.4 to adjust for genotype difference. One participant was excluded from data analysis. [a]T-scores used for analysis. [b]Age effect controlled. [c]Age and sex effects controlled.

© 2023 Braga J et al. *JAMA Psychiatry.*

eTable 2. Difference in Ventral Striatum and Dorsal Putamen TSPO V_T Between Groups Relative to TSPO V_T in Other Brain Regions

Dependent Variable	Independent Variables	Effect of Group[a]
Ventral Striatum TSPO V_T	Group, Prefrontal Cortex TSPO V_T	0.01
Ventral Striatum TSPO V_T	Group, Hippocampus TSPO V_T	0.02
Ventral Striatum TSPO V_T	Group, Anterior Cingulate Cortex TSPO V_T	0.1
Dorsal Putamen TSPO V_T	Group, Prefrontal Cortex TSPO V_T	0.003
Dorsal Putamen TSPO V_T	Group, Hippocampus TSPO V_T	0.05
Dorsal Putamen TSPO V_T	Group, Anterior Cingulate Cortex TSPO V_T	0.1

[a]p-value from analysis of variance with group as predictor of interest and the other region (either prefrontal cortex, anterior cingulate cortex, or hippocampus) as a covariate

© 2023 Braga J et al. *JAMA Psychiatry.*
