# Supplementary material for: Neuroinflammation After COVID-19 With Persistent Depressive and Cognitive Symptoms
Source: JAMA Psychiatry. 2023 May 31;80(8):787–95. doi: 10.1001/jamapsychiatry.2023.1321 (PMC10233457; doi:10.1001/jamapsychiatry.2023.1321)
Supplement: Supplement 2. — Data Sharing Statement [file jamapsychiatry-e231321-s002.pdf]

Data Sharing Statement

Braga. Neuroinflammation After COVID-19 With Persistent Depressive and Cognitive Symptoms. *JAMA Psychiatry*. Published May 31, 2023. doi:10.1001/jamapsychiatry.2023.1321

Data
Data available: Yes
Data types: Deidentified participant data
How to access data: Data collected from the study corresponding to Figure 1, Figure 2 (A to D) will be made available in a Supplementary Appendix 2 at the time of publication. Data for each figure will be provided in deidentified form. For figure 1, data will include an anonymous study id, diagnosis, TSPO VT for each region. For figure 2, data will include the TSPO VT value and associated clinical data.
When available: With publication

Supporting Documents
Document types: None

Additional Information
Who can access the data: The data will be available to anyone without restriction.
Types of analyses: The data will be made available for any purpose.
Mechanisms of data availability: The data will be made available by accessing Supplementary Appendix 2.
